# Influence of a new abutment design concept on the biomechanics of peri-implant bone, implant components, and microgap formation: a finite element analysis

**DOI:** 10.1186/s12903-023-02989-x

**Published:** 2023-05-11

**Authors:** Huimin Nie, Yantai Tang, Yan Yang, Weijie Wu, Wenjuan Zhou, Zhonghao Liu

**Affiliations:** 1 Department of Implant Dentistry, Binzhou Medical University Affiliated Yantai Stomatological Hospital, No. 19, Beima Road, Zhifu District, Yantai, 264000 China; 2 University of Science and Technology Beijing, Beijing, 100000 China

**Keywords:** Dental implant, Abutment design, Principal stress, Von Mises stress, Microgap, Finite element analysis

## Abstract

**Background:**

A new two-piece abutment design consisting of an upper prosthetic component and tissue-level base has been introduced; however, the biomechanical behavior of such a design has not been documented. This study aimed to investigate the effect of a two-piece abutment design on the stress in the implant components and surrounding bone, as well as its influence on microgap formation.

**Methods:**

To simulate the implant models in the mandibular left first molar area, we established nine experimental groups that included three bone qualities (type II, III, and IV) and three implant–abutment designs (internal bone level, tissue level, and a two-piece design). After the screw was preloaded, the maximum occlusal (600 N) and masticatory (225 N) forces were established. Finite element analysis was performed to analyze the maximum and minimum principal stresses on the peri-implant bone; the von Mises stresses in the implants, abutments, bases, and screws, and the microgaps at the implant–abutment, implant–base, and base–abutment interfaces.

**Results:**

For all three loading methods, the two-piece abutment design and bone-level connection exhibited similarities in the maximum and minimum principal stresses in the peri-implant bone. The von Mises stresses in both screws and bases were greater for the two-piece design than for the other connection types. The smallest microgap was detected in the tissue-level connection; the largest was observed at the implant–base interface in the two-piece design.

**Conclusions:**

The present study found no evidence that the abutment design exerts a significant effect on peri-implant bone stress. However, the mechanical effects associated with the base and screws should be noted when using a two-piece abutment design. The two-piece abutment design also had no advantage in eliminating the microgap.

## Background

Mechanical complications causing damage to dental implants or their superstructures have been associated with the design of the implant components and can lead to biological complications such as peri-implant tissue damage. Owing to the connection between implant complications and their components, knowledge of the biomechanical influence of implant–abutment connection design is essential for the long-term stability and success of dental implant treatment [1].

In general, the traditional classification of implant–abutment connections is based on the presence of geometric features on the implant’s coronal surface [2]. The abutment connections are then divided into either external or internal connections. The internal connections have been proven to be more favorable [3]. The connections can be further classified into bone-level (BL) and tissue-level (TL) connections according to the implant platform’s alignment with the soft tissue. Both designs reportedly correlate with stress distribution in the peri-implant bone and implant components and with the microgap at the implant–abutment interface (IAI) [4–7]. The stress around the peri-implant bone is responsible for marginal bone loss [8]; implant component overloading can cause mechanical complications, such as screw loosening or implant and abutment fracture; and the microgap is associated with bacterial infiltration, which may lead to biological complications [9].

The effects of different implant–abutment connections on the implant components and surrounding bone have been evaluated in several studies comparing strains and stresses among different abutment connection types (platform switching, external hexagon, and Morse taper). These studies have confirmed the advantages of the Morse taper connection designs [10–12]. Other studies have divided the implant–abutment connection into BL and TL types, reporting that TL connection components have lower stress values and may be preferable for maintaining the marginal bone level [6, 13, 14]. Most studies have concluded that the conical connection [15, 16] and TL connection [4, 7] cause the fewest microgaps.

TL connection designs have been proven to have more advantages for stress distribution and microgap minimization; however, BL connections are used in a large proportion of clinical applications owing to their aesthetic advantages [17]. To minimize implant component-related technical or biological complications, implant manufacturers have improved design concepts by using implant–abutment connections, which have both advantages and disadvantages. Nobel Biocare developed the On1 concept that uses an internal connection, a two-piece (TP) abutment design comprising an upper prosthetic component, which can be replaced, and a tissue-level base attached immediately after implant placement. This design protects soft tissue health without requiring removal of the base portion during impression and has been associated with a decrease in the bone margin level at implant sites [18–21]. However, such a design has an additional upper short screw that is more fragile and requires two connection interfaces (i.e., the implant–base interface (IBI) and the base–abutment interface (BAI), which may lead to more interface-related complications. To the best of our knowledge, the influence that such a design has on the stress of implant components and surrounding bone, as well as microgap formation, has not been documented.

Bone quality has been shown to correlate with the implant survival rate, with a higher failure rate for implants exhibiting low bone quality [22, 23]. To further evaluate the TP abutment design concept, finite element analysis (FEA) was applied to simulate the maximum occlusion and mastication for three bone qualities. The present study aimed to address the following questions: 1) Does the TP abutment design concept affect stress distribution in the implant components and surrounding bone? 2) How do of different implant–abutment connections influence microgap formation?

## Materials and methods

**Fig. 1:**
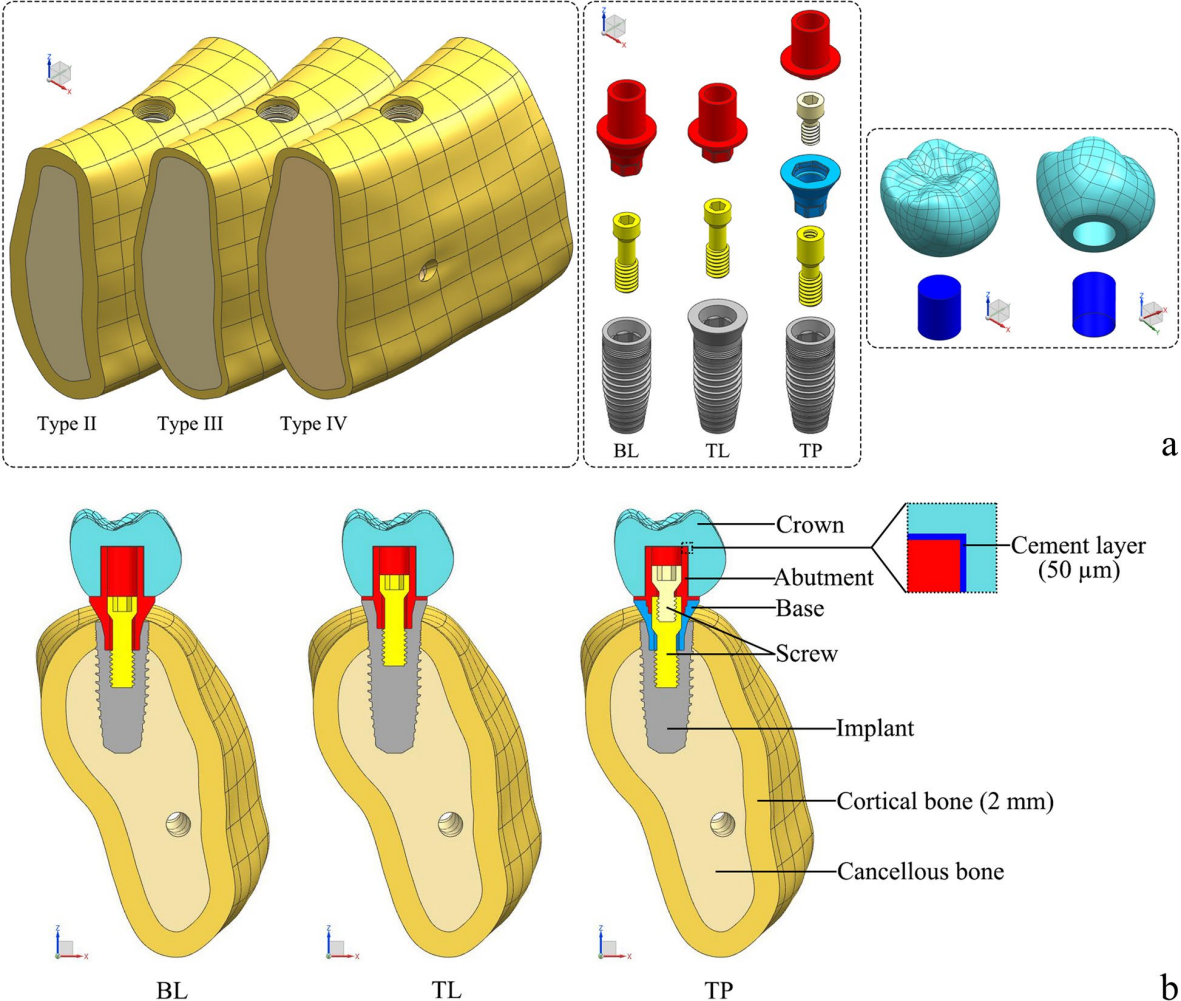
Finite element model structures. **a** Unassembled models; **b** Assembled models

**Fig. 2:**
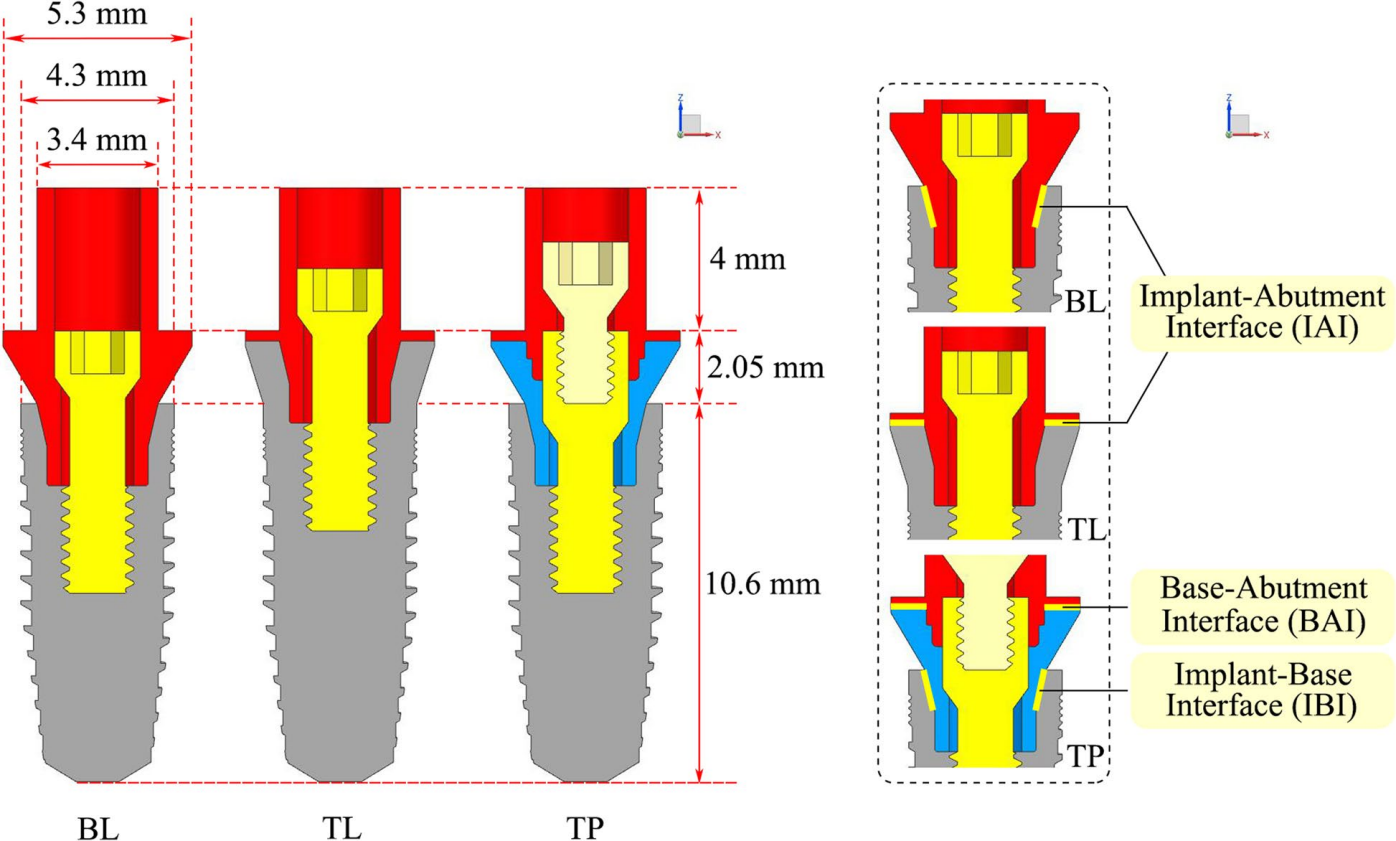
Dimensions of three types of implant components and contact surfaces for the analysis of microgap formation. Note: The length and diameter of the implant were 10.6 mm and 4.3 mm; the diameter at the abutment platform was 5.3 mm; the height of the part above the abutment platform was 4 mm, and the diameter was 3.4 mm; the total transmucosal height was 2.05 mm

A total of nine three-dimensional finite element models were established (Table 1). Two parameters were considered: bone quality (types II, III, and IV) and the implant–abutment design (BL, TL, or TP connection design). All models consisted of the alveolar bone in the mandibular first molar region and the implant complex (Fig. 1). The simulation of the alveolar bone was performed by reconstructing the cone beam computed tomography data using commercial modeling software (Mimics 21.0, Materialise Group; Geomagic Wrap 2017, 3D Systems). The bone tissue comprised cancellous bone in the center, surrounded by 1 or 2 mm of cortical bone (only type II bone had a cortical bone thickness of 2 mm). The nerve canal was reconstructed using Boolean subtraction. The implant complex was simulated based on the Nobel system, including the following components: the implant, abutment, base, screw, cement layer, and crown. The cement layer thickness was set at 50 µm. Figure 2 shows the dimensions of the three types of implant components. For each system, the same length and diameter were used for both the implant and abutment, although different types of implants, abutments, and screws were used.

**Table 1:** Number of elements and nodes used for each model

Connections	Bone quality	Groups	Elements	Nodes
Bone-level	Type II bone	BL-II	2,251,932	432,856
	Type III bone	BL-III	2,262,372	433,870
	Type IV bone	BL-IV	2,262,372	433,870
Tissue-level	Type II bone	TL-II	2,346,933	451,495
	Type III bone	TL-III	2,357,373	452,509
	Type IV bone	TL-IV	2,357,373	452,509
Two-piece design	Type II bone	TP-II	2,473,686	476,947
	Type III bone	TP-III	2,484,126	477,961
	Type IV bone	TP-IV	2,484,126	477,961

Table 1 lists the number of elements and nodes in each model. The material properties of the model components, based on previous studies, are summarized in Table 2 [24–30]. All materials defaulted as homogenous, linearly elastic, and isotropic. To calculate the microgap, the interfaces between the implant, base, abutment, and screws were defined as “contacts”. The coefficient of friction was set to 0.3. The bone–implant interface was defined as a “tie” to symbolize complete osseointegration. In each group, fixation constraints were placed on the mesial and distal sides of the cortical and cancellous bone.

**Table 2:** Material properties of the finite element model

Material	Modulus of elasticity (MPa)	Poisson’s ratio	References
Pure titanium (implant)	110,000	0.33	[24, 25]
Titanium alloy (base, abutment, all screws)	110,000	0.33	[24, 25]
Lava Zirconia (crown)	210,000	0.3	[26, 27]
Cement	6400	0.27	[27]
Cortical bone	13,700	0.3	[28]
Type II cancellous bone	5500	0.3	[29, 30]
Type III cancellous bone	1600	0.3	[29, 30]
Type IV cancellous bone	690	0.3	[29, 30]

**Fig. 3:**
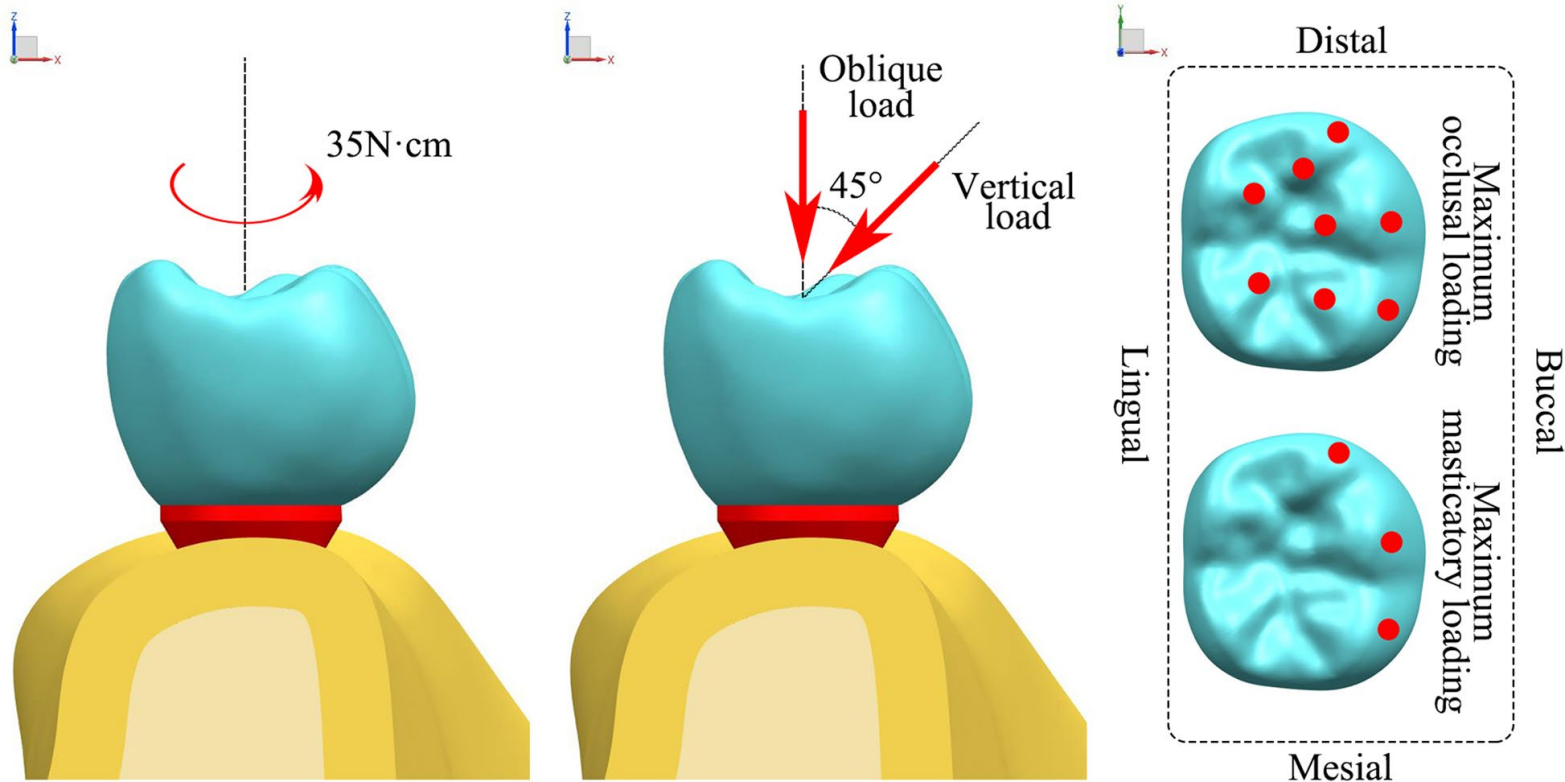
Loading conditions

The simulation consisted of two steps (Fig. 3). First, preloading was applied to simulate a tightening torque of 35 N-cm on the screw. Second, external loading was applied to the crown to simulate the maximum occlusal and masticatory loadings [31]. The maximum occlusal loading was applied in the vertical direction. A force of 600 N was applied at eight occlusal points: three on the buccal bevel of the buccal tip, three on the lingual bevel of the buccal tip, and two on the buccal bevel of the lingual tip. The maximum masticatory loading was applied in the vertical and oblique directions (45° to the tooth axis). A force of 225 N was applied to three occlusal points on the buccal bevel of the buccal cusp.

The FEA was completed using ABAQUS 2021 software (Dassault SIMULIA, France). The maximum and minimum principal stress values were calculated to evaluate the tensile and compressive stresses on the peri-implant bones. The tensile and compressive stresses were distinguished by their positive and negative values, respectively. The von Mises stress peaks were used to analyze the biomechanical behavior of the implants, abutments, bases, and screws. The microgap peaks for the IAI, IBI, and BAI were also calculated (Fig. 2).

## Results

### Maximum and minimum principal stresses

**Fig. 4:**
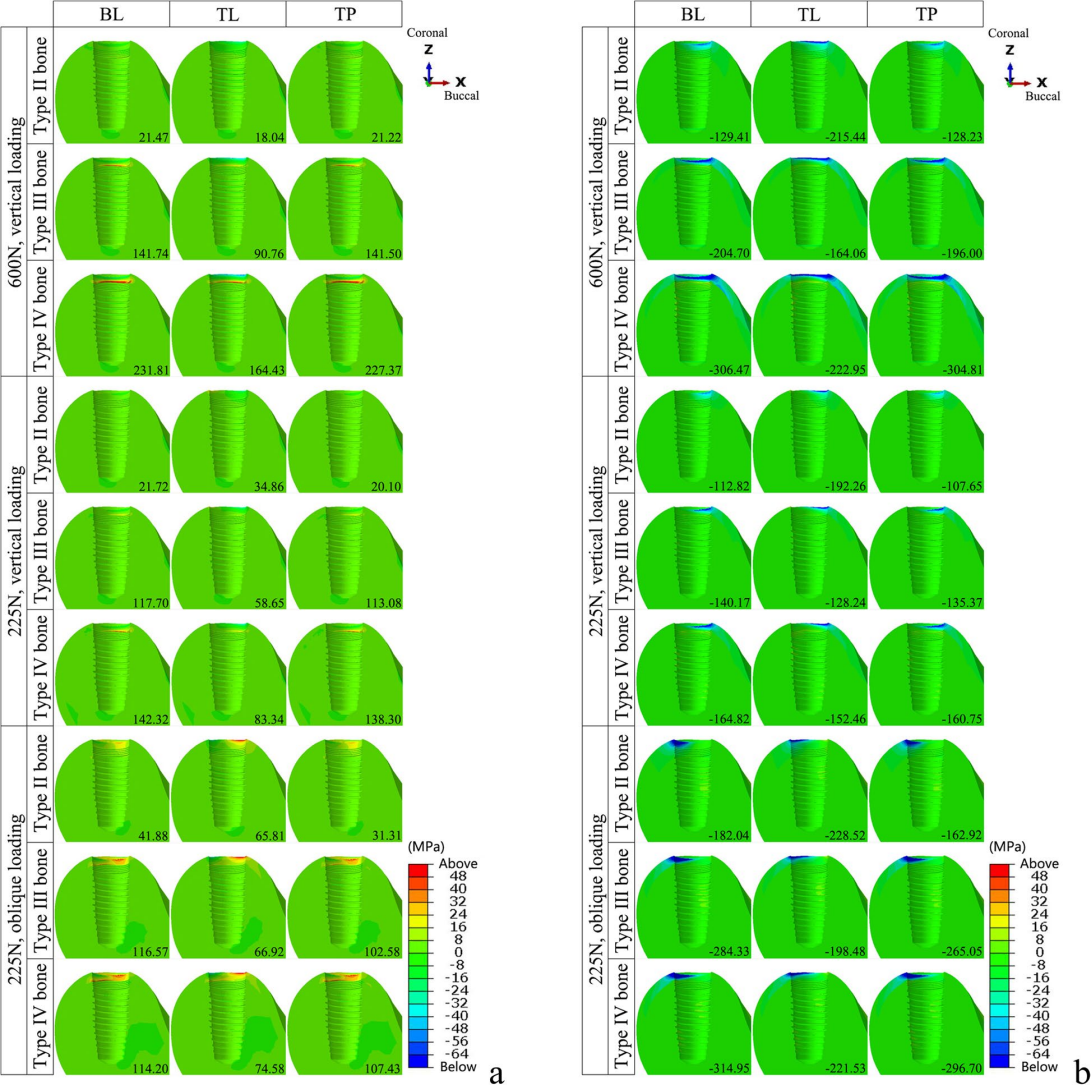
The distribution of the maximum and minimum principal stresses on the peri-implant bone in the models with different connection designs and bone qualities for three loading conditions. **a** Maximum principal stress; **b** Minimum principal stress

**Fig. 5:**
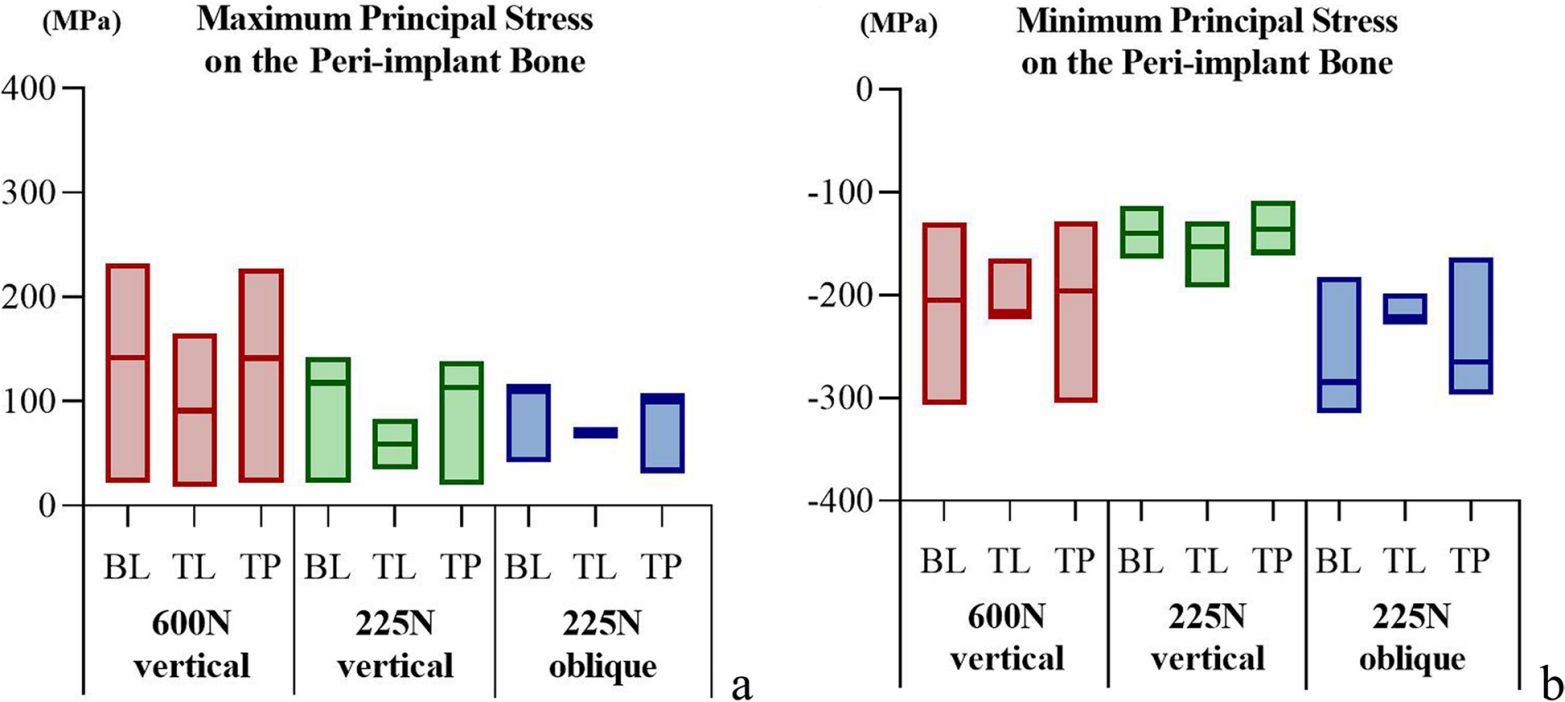
The peak values for maximum and minimum principal stress on the peri-implant bone in the models with different connection designs under three loading conditions. **a** Peak values for maximum principal stress; **b** Peak values for minimum principal stress

**Fig. 6:**
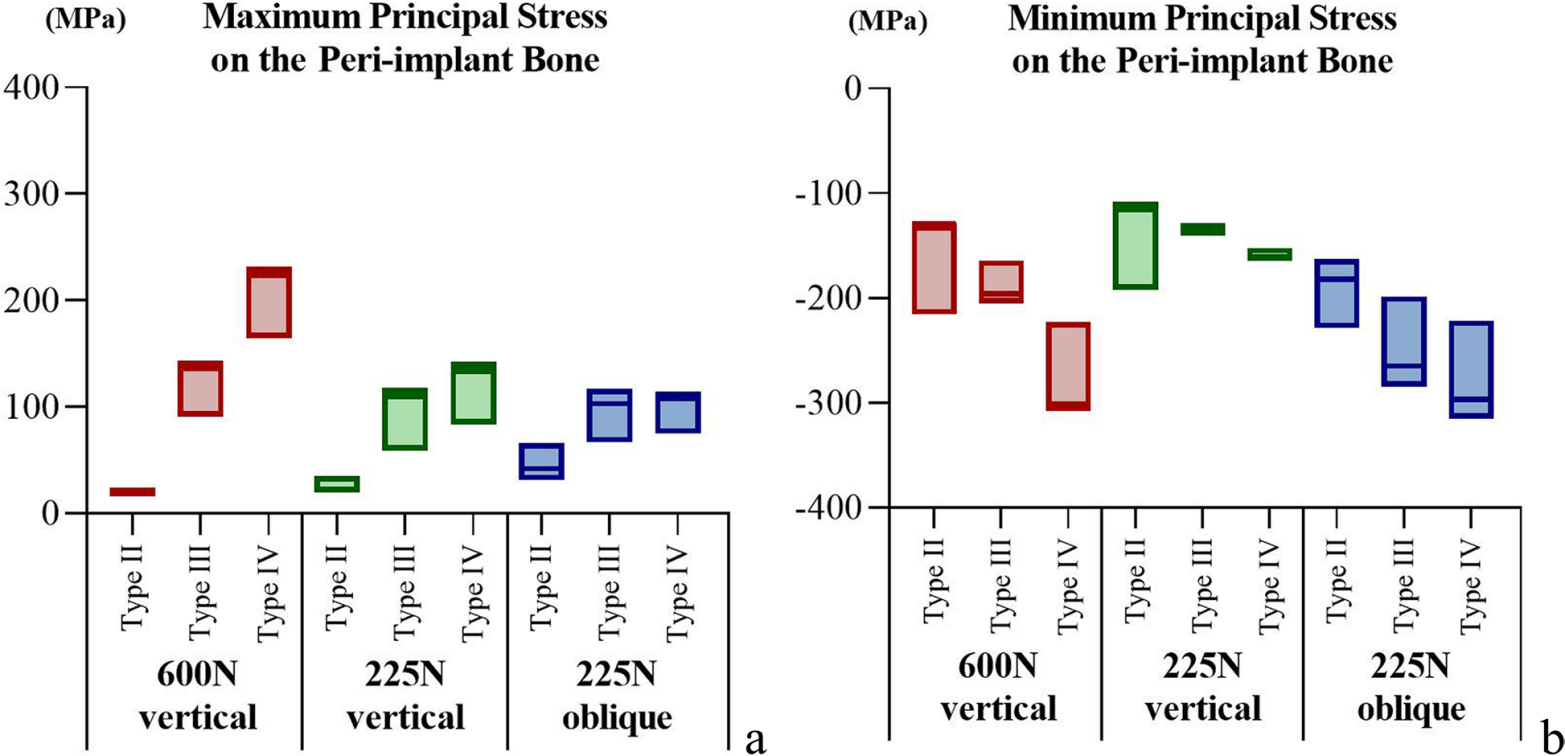
The peak values for maximum and minimum principal stress on the peri-implant bone in the models with different bone quality under three loading conditions. **a** Peak values for maximum principal stress; **b** Peak values for minimum principal stress

The maximum and minimum principal stress analysis revealed that the tensile and compressive stresses were mainly distributed in the cortical bone. In the models with varied implant–abutment connection designs, the stress distribution in the peri-implant bone was essentially the same. The stress peaks in the TP model were similar to those in the BL model. The effect of the different bone qualities was greater than that of the implant–abutment connection designs. The stress distribution range and peak value increased with decreasing cortical bone thickness and cancellous bone density. At a load of 225 N, the oblique force resulted in a greater range of stress distribution and peak compressive stress (Figs 4, 5 and 6).

### Von Mises stresses

**Fig. 7:**
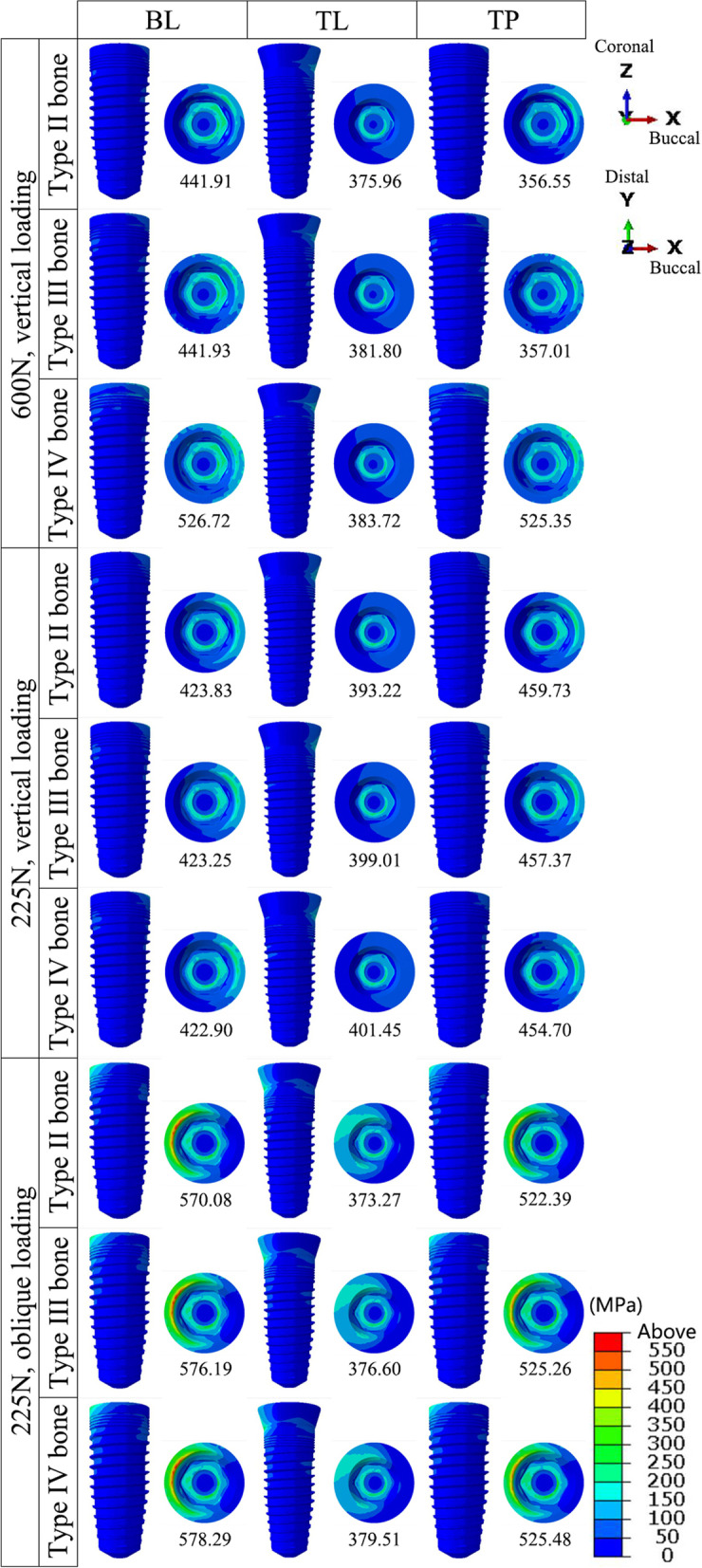
The distribution of the von Mises stress in the implants in the models with different connection designs and bone qualities for three loading conditions

In the implants, the von Mises stresses were concentrated mainly at the internal thread and in the implant neck, above the bone level in the TL implant and below the bone level in the BL and TP models. The minimum stress in the implants occurred in the TP-II group (356.55 MPa), while the maximum stress occurred in the BL-IV group (578.29 MPa). The peak stresses in the TL implants were smaller than those in the BL implants under all three loading conditions (Figs. 7 and 10a).

**Fig. 8:**
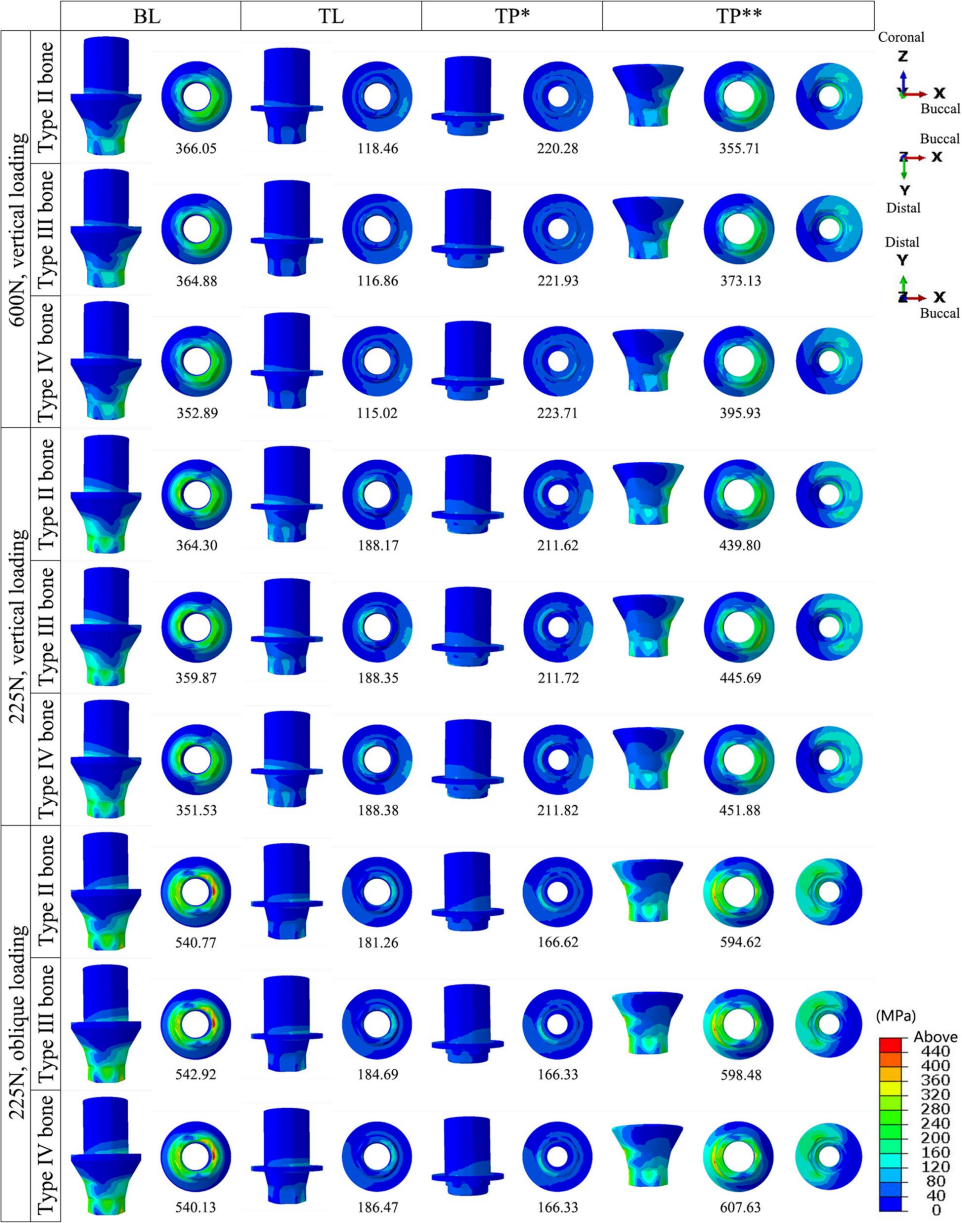
The distribution of the von Mises stress in the abutments and bases in the models with different connection designs and bone qualities for three loading conditions. * abutments; ** bases

The stresses in the abutments and bases were mainly dispersed in the connection region. The stresses in the abutments of the TL and TP models exhibited smaller peaks and a more even distribution. The lowest stresses were observed in the TL-IV group of abutments (115.02 MPa), whereas the highest were observed in the TP-IV group of bases (607.63 MPa). For all loading conditions, the base in the TP model was subjected to the greatest peak stresses among all connection designs, followed by the BL model (Figs. 8 and 10b).

**Fig. 9:**
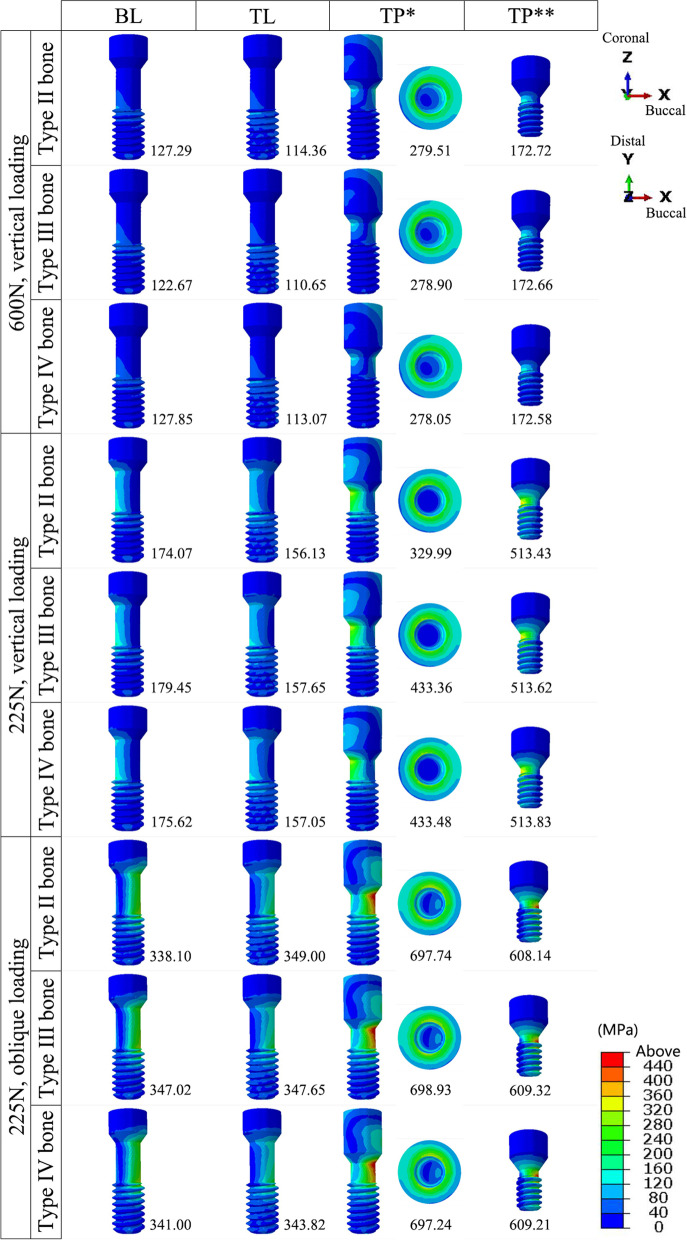
The distribution of the von Mises stress in the screws in the models with different connection designs and bone qualities for three loading conditions. * conventional screws; ** upper short screws

**Fig. 10:**
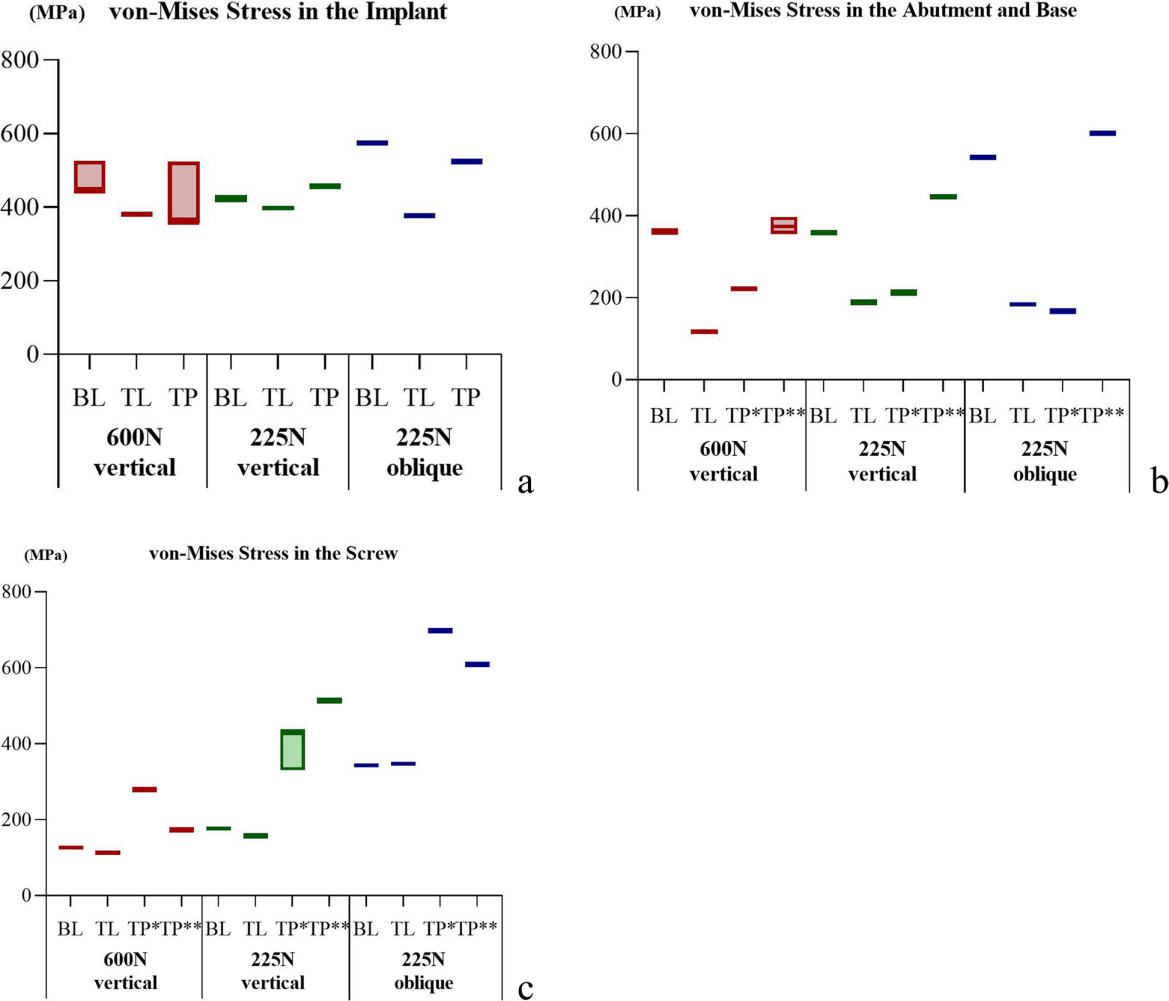
The peak values for von Mises stress in the implant components under three loading conditions. **a** Peak values for von Mises stress in the implants; **b** Peak values for von Mises stress in the abutments and bases (* abutments; ** bases); **c** Peak values for von Mises stress in the screws (* conventional screws; ** upper short screws)

The stresses in the screws were mainly distributed in the unthreaded bars and were also present in the internal threads of the standard screws in the TP model. Higher stresses were observed for the two screws in the TP model than for the screws in the BL and TL models. The TL-III group of screws had the lowest von Mises stress value (110.65 MPa), and the TP-III group of conventional screws had the highest value (698.93 MPa) (Figs. 9 and 10c).

The bone quality had no effect on the distribution or peak of von Mises stresses in the implants, abutments, bases, or screws. The implants and abutments in the TL connection, as well as the abutments in the TP design, were less affected by the various loadings. The other components, however, experienced elevated stress levels as a result of the oblique loading.

### Microgaps

**Fig. 11:**
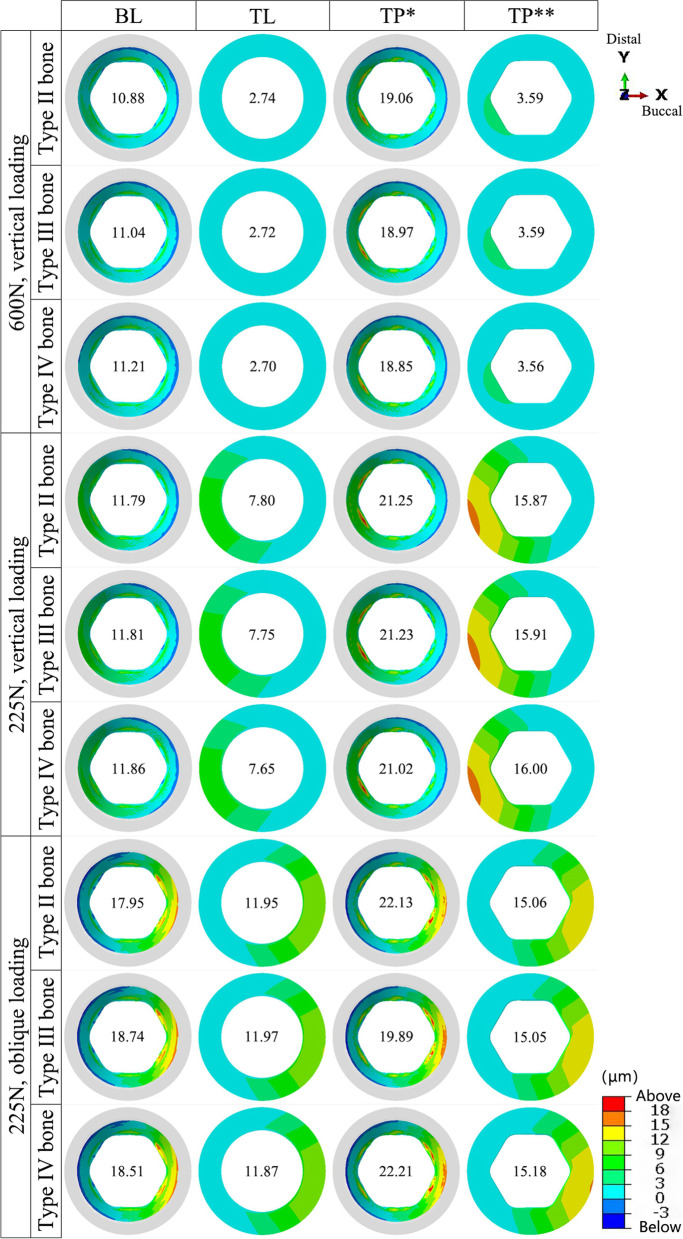
The distribution of the interface microgap in the models with different connection designs and bone qualities for three loading conditions. * implant–base interface; ** base–abutment interface

**Fig. 12:**
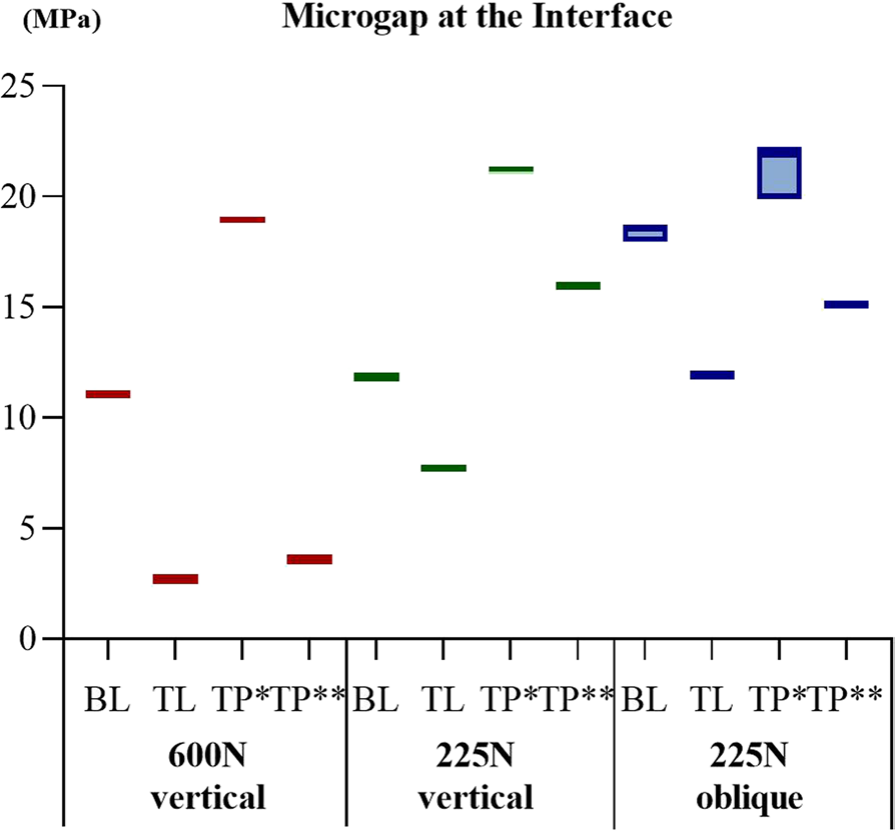
The peak values at the interface microgap under three loading conditions. * implant–base interface; ** base–abutment interface

For the BL connection model, the microgap distribution at the IAI was comparable to the distribution at the IBI in the TP model. For the TL connection model, the distribution at the IAI was similar to the distribution at the BAI in the TP model. Under all three loading conditions, the microgap at the IAI for the TL connection model was the smallest, with a minimum value of 2.70 µm (TL-IV group); the microgap at the IBI for the TP connection was the largest, with a minimum value of 22.21 µm (TP-IV group). The size of the microgap was nearly independent of the bone quality. The microgap at the IAI interface for the BL and TL connection types was significantly widened under the 225 N oblique load (Figs. 11 and 12).

## Discussion

The TP abutment design preserves the bone interface and connective tissue during the healing period and all restorative operations, avoiding the drawbacks associated with “one abutment one time” or TL implants [20]. However, reports on this design’s biomechanical effects have been limited. In this study, the FEA technique was used to evaluate the stresses in the implant components and surrounding bone as well as the microgap at the connection interface in a TP model. Our results indicated that the TP and BL connections had similar distributions for the maximum and minimum principal stresses in the peri-implant bone; however, the von Mises stress peaks in the bases and screws and the microgap peak at the IBI were significantly larger for the TP connection than for other connection types.

Occlusal overload is among the causes of peri-implant bone resorption. Ideally, the implant–abutment connection design should prevent the transmission of excessive loads to the surrounding bone tissue. Araki and Avag et al. [13, 14] compared the difference in stress and strain in bone tissue between the BL and TL implants. Since the TL implant platform was located above the bone surface, the stresses were all concentrated above the alveolar bone, and the stresses in the bone tissue were much less. In contrast, we did not observe significant differences in the peak tensile or compressive stresses in the bone tissue among the three connection designs. This may be because we utilized loading conditions different from those in the previous study, in which loading forces ranged from 50 to 150 N.

The various bone qualities had a greater effect on the maximum and minimum principal stresses than the implant–abutment connection design. Lee et al. [6] simultaneously analyzed the biomechanical effects of different implant diameters, connection types, and bone densities. In their study, bone density was identified as the most influential factor for bone tissue strain, with low-density bone leading to greater principal strain peaks (*P* < 0.001). This present study likewise found that the lowest principal stress was always observed in type II bones because they have a significantly greater elastic modulus than bone types III and IV. The greater the elastic modulus, the greater the resistance to compression [29]. Although patients with poor bone quality should be managed with care in clinical practice, these results suggest that bone quality hardly affected the stresses in the implant components and the microgap at the interfaces. Lemos et al. [11] evaluated the effect of vertical bone loss in normal and osteoporotic bone using 3D FEA. Their results also showed that, unlike progressive bone loss, osteoporotic bone affected only the microstrain in the trabecular bone but not the stress in the implants or prosthetic components.

However, the von Mises stress peaks in the implant components differed among the implant–abutment connection designs in our study. Lee et al. [5] reported that stresses in the implant, abutment, and screw were greater for the BL model than the TL model under both vertical and oblique loading conditions. In particular, the stress value of the BL-connected abutment was 3.5 times higher than that of the TL-connected abutment. Similar results have been obtained in other studies [6, 7, 13]. This may cause the better crown-to-root ratio of the TL model when compared with those for the BL and TP connection designs, allowing the TL implant to provide better leverage and more mechanical advantage than the BL implant. For abutments, the load was transferred from the abutment to the BL implant, relying exclusively on the contact surface between the implant and abutment. However, the wide neck of the implant supported the forces from the crown in the TL connection. Some forces were delivered directly to the implant platform, reducing the stress on the abutment [5]. The base of the TP model included a wide platform, similar to the TL implant, allowing direct contact with the crown and helping distribute the stress in the abutment. However, as it can only be transferred to the implant through the connection interface, this resulted in the highest von Mises stress in the base. Additionally, the two screws in the TP model had greater stress under the same loading, even 2–3 times greater than in the BL and TL models. Pumnil et al. [32] analyzed the stress distributions of four different personalized abutments using static FEA, including a two-piece personalized abutment made of a titanium substrate bonded to a zirconia abutment. The authors reported that the titanium base dispersed the stresses in the implant and observed stress concentrations in the screws, in accordance with the findings of the current study. Thus, addressing the mechanical challenges related to the base and screw in the TP connection design is important.

The implant–abutment connections exerted a significant effect on the microgap. Both in vivo and in vitro studies have shown that no connection designs can completely eliminate microgaps and microleakage, but can only minimize them [2]. Zipprich et al. [33] used X-ray imaging to measure the microgap at the IAI of 20 different implant systems for simulated loading up to 200 N, employing conical and flat connections of 0–18.6 and 4.8–42 µm, respectively. Rack et al. [34] observed a microgap of 22 µm under a load of 100 N using high-resolution radiography combined with hard X-ray synchrotron radiation. In this study, the microgap was 2.70–22.21 µm, consistent with the results detected by X-ray imaging. TL connections had the smallest interface microgap because the TL implant neck was larger, and the forces were directed more toward the model’s interior [3, 7]. In contrast, the BL connection model did not have a large neck, and the forces directed to the outside of the model had a greater leveraging effect, leading to a larger microgap. Similarly, the diameter of the base was larger than that of the implant in the TP model, resulting in a smaller microgap at the BAI than at the IBI. Larger microgaps may lead to micromotion and increase the possibility of screw loosening or fracture [35]. Therefore, stomatologists should advise patients with TP abutments to be mindful of oral hygiene and to make regular hospital visits.

Eskitascioglu et al. [36] used FEA to investigate the effect of one to three different loading positions on an implant-fixed denture and surrounding bone stress distribution, observing that the implant and surrounding bone are under excessive stress when force is applied to a single site. Therefore, eight occlusal points were used in this investigation, based on the actual occlusal position during functional movements [31]. The reported average maximum occlusal force in the molar region is 545.7 N; the maximum masticatory force is approximately 37–40% [31, 37]. Our study simulated maximum occlusal and masticatory forces using loadings of 600 N and 225 N (approximately 37% of 600 N). This loading condition was considered the most extreme case. In this study, the implant material was assumed to be pure titanium with a fracture strength of 690 MPa; the abutments, bases, and screws were titanium alloys with a fracture strength of 920 MPa [38]. The analysis showed that all the materials were within the limits under these extremely loading conditions.

Loading conditions are known to influence the stress and microgap [39, 40]. Kim et al. [7] applied a load of 200 N in five directions (0°, 15°, 30°, 45°, and 60°) in order to evaluate the relationship between the loading direction and stress values. In their study, the lowest stress level was observed at a load inclined at 15°, which can be explained by the centrally oriented loading direction and the low torque of the implant and abutment. When the loading angle was increased to 30°, 45°, and 60°, the stress in the implant increased accordingly. The present study likewise found that an oblique force of 45° increased the leverage effect, resulting in greater stresses and microgaps at the same loading of 225 N. Therefore, clinicians should reduce the cusp inclination and minimize the interference of oblique forces when shimming the occlusal contact of the implant prosthesis.

This study had several limitations. First, the finite element model used in our analysis was simplified. For example, while modeling the various bone qualities, only the thickness of cortical bone and the mechanical properties of cancellous bone were altered; their porosity was not considered. This study also assumed that each component was isotropic and that the implant was attached completely to the bone. Although these simplifications sped up the computation process, these assumptions may not reflect clinical reality. Nonetheless, while the exact values among our results may have been affected, these considerations are unlikely to alter the general trend of the findings.

## Conclusions

Within the scope of this study, the following conclusions can be drawn:

1. The present study found no evidence that different abutment designs make a significant difference on peri-implant bone stress.
2. The mechanical effects associated with the base and screws should be noted when using a TP abutment design.
3. TL connections ensure the smallest microgaps under maximum occlusal or masticatory force, although the TP abutment design had no advantages in eliminating the microgaps.

## Data Availability

All data were calculated by the software itself. The datasets used and/or analyzed during the current study are available from the corresponding author on reasonable request.

## Abbreviations

BAI: Base-abutment interface
BL: Bone-level
IAI: Implant-abutment interface
IBI: Implant-base interface
TL: Tissue-level
TP: Two-piece
